# Fe-Co Co-Doped 1D@2D Carbon-Based Composite as an Efficient Catalyst for Zn–Air Batteries

**DOI:** 10.3390/molecules29102349

**Published:** 2024-05-16

**Authors:** Ziwei Deng, Wei Liu, Junyuan Zhang, Shuli Bai, Changyu Liu, Mengchen Zhang, Chao Peng, Xiaolong Xu, Jianbo Jia

**Affiliations:** 1 Jiangmen Key Laboratory of Synthetic Chemistry and Cleaner Production, School of Environmental and Chemical Engineering, Carbon Neutrality Innovation Center, Wuyi University, Jiangmen 529020, China; dengziwei22@163.com (Z.D.); zhangjunyuan0911@163.com (J.Z.); wyuchembsl@126.com (S.B.); wyuchemcyliu@126.com (C.L.); nj_zmc@126.com (M.Z.); wyuchempc@126.com (C.P.); 2 Jiangmen Customs District Technology Center, Jiangmen 529020, China; worklius@163.com

**Keywords:** bifunctional N-doped carbon, mesoporous structure, oxygen reduction reaction, oxygen evolution reaction, zinc–air battery

## Abstract

A Fe-Co dual-metal co-doped N containing the carbon composite (FeCo-HNC) was prepared by adjusting the ratio of iron to cobalt as well as the pyrolysis temperature with the assistance of functionalized silica template. Fe_1_Co-HNC, which was formed with 1D carbon nanotubes and 2D carbon nanosheets including a rich mesoporous structure, exhibited outstanding oxygen reduction reaction (ORR) and oxygen evolution reaction (OER) catalytic activities. The ORR half-wave potential is 0.86 V (vs. reversible hydrogen electrode, RHE), and the OER overpotential is 0.76 V at 10 mA cm^−2^ with the Fe_1_Co-HNC catalyst. It also displayed superior performance in zinc–air batteries. This method provides a promising strategy for the fabrication of efficient transition metal-based carbon catalysts.

## 1. Introduction

The immoderate utilization of fossil fuels can cause the serious environmental deterioration problems such as global warming. It is imperative to accelerate the application of new energy resources and the development of clean energy technologies [1,2]. Currently, metal–air batteries and fuel batteries are the most effective means of reducing energy shortage [3,4,5]. Nevertheless, they require efficient bifunctional catalysts for both the oxygen reduction reaction (ORR) at the cathode and the oxygen evolution reaction (OER) at the anode [6,7]. There are mainly Pt, Ru, and Ir catalysts in the current market. Unfortunately, they usually do not possess the bifunctional catalytic activity for both ORR and OER at the same time. In addition, the high cost and poor stability limit the development and application of batteries on the large scale [8,9]. Therefore, it is crucial to develop efficient, low-cost, and durable bifunctional catalysts for ORR and OER at the same time [10,11].

Since cobalt phthalocyanine exhibits some ORR electrocatalytic activity [12], various catalysts composed of transition metals incorporated into nitrogen-doped carbon matrices (M-N-Cs, especially Fe-N-C and Co-N-C) have been studied extensively [13,14,15,16]. However, most of the monometallic M-N-Cs displayed an unsatisfactory performance as the bifunctional catalysts for ORR and OER [17,18]. Thus, there is a great challenge to develop efficient bifunctional ORR/OER electrocatalysts. A reasonable solution would be engineering the N-doped carbon matrix with bimetallic sites so that the performance can be tuned accordingly [19,20,21]. In addition, numerous studies have shown that the catalyst structure has a significant impact on its performance. For example, the modified zeolitic imidazolate frameworks (SiO_2_@Fe-ZIF-8/ZIF-67) was designed to prepare the atomically dispersed Fe and Co doping 3D nitrogen-doped carbon nanosheets (CNSs), which exhibited excellent electrochemical performance with a ORR/OER potential gap (the potential difference (∆E) of half-wave potential (E_1/2_, V vs. reversible hydrogen electrode (RHE)) for ORR and potential at the current density of 10 mA cm^−2^ (E_j=10_, V vs. *i*R-corrected RHE) for OER, which is an important indicator for evaluating the bifunctional catalyst of 0.80 V [22,23]. Recently, ZnCo bimetallic sites were incorporated in hierarchical N-doped carbon via a one-step pyrolysis method, and displayed excellent ORR catalytic activities [24]. By integrating 1D carbon nanotubes (CNTs) and 2D CNSs, the problem of agglomerating and stacking 2D materials can be solved, and the surface area and chemical stability were improved [24,25,26]. The integrated 1D@2D layered structure has enhanced surface area, better operational stability, and higher conductivity [27].

Herein, the Fe-Co bimetal co-doped N containing the carbon composite (Fe_x_Co-HNC-T) was prepared with modified silicon nanospheres as sacrificial template. Through using Fe@ZIF-8/ZIF-67, 1D CNTs with diameter of ~40 nm was generated on the surface of silicon nanospheres, and 2D CNSs with high specific surface area was obtained after etching of the silicon nanosphere templates. The main goal is to provide an efficient method for the development of non-precious metal electrocatalysts and to expand the application of zinc–air batteries in the near future. In the final structure, nitrogen-doped carbon was arranged in a hierarchical manner with abundant mesoporous 1D nanotubes and 2D nanosheets with Fe_1_Co-HNC-1000 showing excellent ORR/OER bifunctional catalytic activity. When the prepared composite was applied as the cathodic catalyst in the zinc–air battery, it exhibited an excellent open circuit voltage (1.660 V), high power density (94.9 mW cm^−2^), and good cycling stability (67 h). Compared to previous catalysts for zinc-air battery cathodes, it displayed the improved performances in rechargeable zinc-air batteries [28]. These results indicate that Fe_1_Co-HNC-1000 is an ideal bifunctional electrocatalyst for future zinc-air batteries, and provide an important guidance for further understanding and designing bifunctional catalysts.

## 2. Results and Discussion

### 2.1. Structural Characterization

**Scheme 1:**
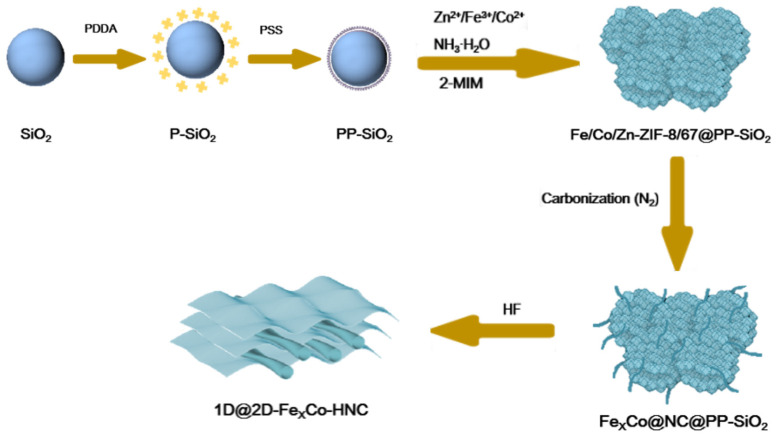
Illustration for synthesis of Fe_x_Co-HNC-T.

**Figure 1:**
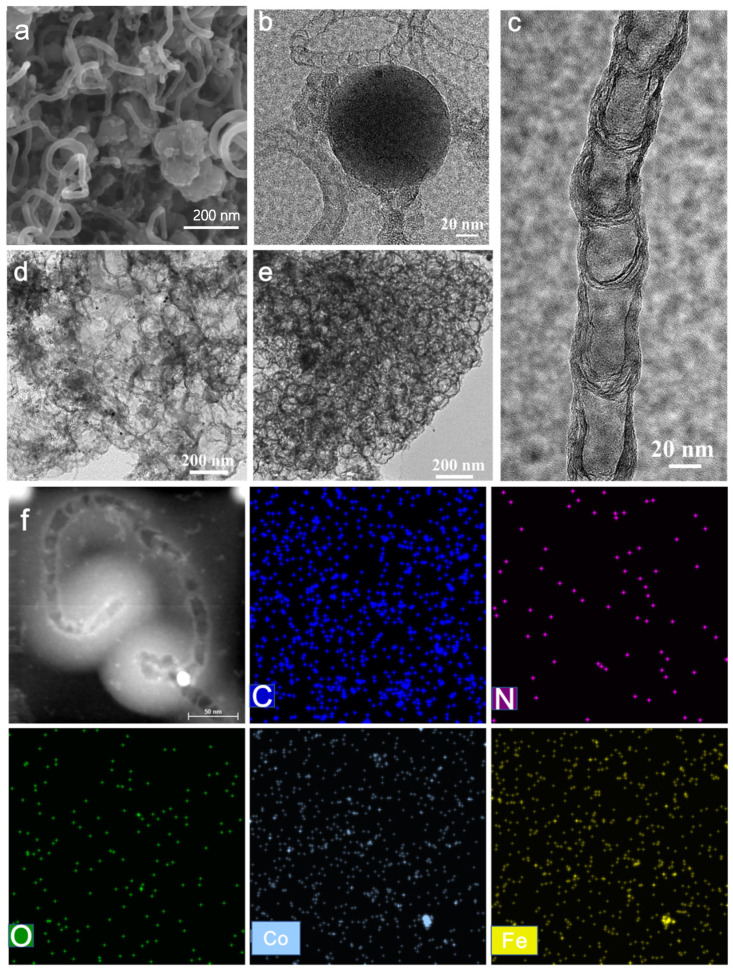
(**a**) SEM images of Fe_1_Co-HNC-1000 (un-etched), TEM images of (**b**) Fe_1_Co-HNC-1000 (un-etched), (**c**) Fe_1_Co-HNC-1000, (**d**) Fe_1_Co-HNC-900, (**e**) Fe_1_Co-HNC-1000, and (**f**) HAADF mappings of Fe_1_Co-HNC-1000.

Scheme 1 shows the synthesis process of the Fe_x_Co-HNC-T composite which was formed by 1D CNTs and 2D CNSs. In the first step, reversed-phase microemulsion was selected to prepare the silica nanosphere template with particle size of ~80 nm, as shown in Figure S1a. Afterward, the positively charged PDDA which can be transferred to the surface of the SiO_2_ nanospheres through electrostatic interaction was added, and the resulting positive charge surface can be obtained (P-SiO_2_). Next, the negatively charged PSS was chosen to modify the P-SiO_2_ nanospheres to get a negative charge surface (PP-SiO_2_, Figure S1b). Positively charged substances, such as metal ions, can be absorbed on the PP-SiO_2_ surface conveniently. Therefore, the electrostatic adsorption capacity of the resulting PP-SiO_2_ nanospheres can be enhanced. PP-SiO_2_ was dispersed in methanol uniformly, followed by the addition of Zn, Co, and Fe salts, and mixed thoroughly with stirring. In the presence of ammonia, metal ions can form corresponding metal hydroxides (MOH) by hydrolysis under alkaline condition. These metal hydroxides can adhere to the surface of the PP-SiO_2_ nanospheres, the corresponding solution color changed from purple to brown. Subsequently, a methanol solution containing 2-methylimidazole ligand was added, resulting in the successful synthesis of Fe/Co/Zn-ZIF-8/ZIF-67@PP-SiO_2_. In contrast, the prepared Fe/Co/Zn-ZIF-8/ZIF-67 exhibits an uneven polyhedral structure in the absence of ammonia (Figure S1c). Therefore, Fe/Co/Zn-ZIF-8/ZIF-67 was able to grow uniformly on the surface of SiO_2_ nanospheres in the presence of ammonia. In addition, the product size was smaller than 50 nm in the presence of ammonia (Figure S1d), indicating that the growth of ZIF-8/ZIF-67 was limited under the given conditions. Figure 1a displays that there are lots of 1D CNTs on the silica nanosphere template. However, the 1D CNTs was agglomerated in the sample generated with the unmodified SiO_2_ template (Figure S2a). The prepared Fe/Co/Zn-ZIF-8/ZIF-67@PP-SiO_2_ were then annealed in nitrogen atmosphere and acid-washed to remove the silica template. The final Fe_x_Co-HNC was obtained with rich 1D CNTs and 2D CNSs structure. Interestingly, compared with Co-HNC, Fe_x_Co-HNC exhibits a uniform CNT structure which can be attributed to the introduction of Fe as the efficient catalyst for the growth of CNTs (Figure S2b,c). Such a structure should be good for improving the structural stability and electronic conductivity.

Based on the SEM image (Figure 1a), 1D CNTs was generated around the silica template. The unetched silica template presented circular shadows (Figure 1b). After acid etching of the sample, bamboo-like CNTs can be observed clearly (Figure 1c). At the same time, CNSs was formed after removing PP-SiO_2_ templates, led to 1D@2D heterostructure of the prepared composites (Figure 1d,e). The formation of 1D@2D heterostructure should be ascribed to the presence of specific PP-SiO_2_ template. In addition, it was found that the sample prepared at 900 °C (Fe_1_Co-HNC-900) generated more CNTs than that prepared at 1000 °C (Fe_1_Co-HNC-1000). However, Fe_1_Co-HNC-1000 displayed more CNSs than that of Fe_1_Co-HNC-900. The unique 1D@2D heterostructure had a greater number of active sites, which should be more conducive to catalytic reaction. In addition, the mapping analysis of Fe_1_Co-HNC-1000 was performed. The result demonstrated the prepared composite was composed of C, N, O, Fe, and Co, indicating that the Fe-Co bimetallic doped N-contained carbon composite was prepared successfully.

**Figure 2:**
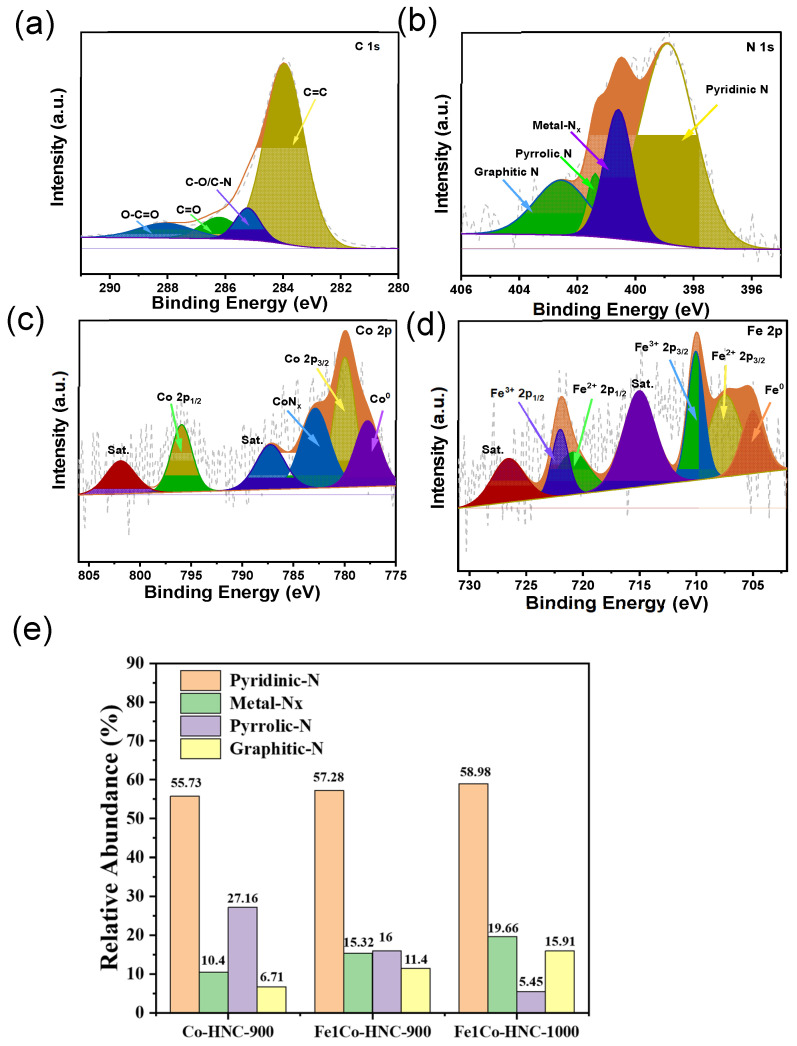
High-resolution XPS spectra of Fe_1_Co-HNC-1000 (**a**) C 1s, (**b**) N 1s, (**c**) Co 2p, and (**d**) Fe 2p. (**e**) Chemical states of N elements in Fe_1_Co-HNCs prepared at different temperatures according to the N1s narrow-scan spectrum.

The crystalline structures of the prepared composites were then characterized using the XRD analysis. It is evident from Figure S3 that Co-HNC-900, Fe_1_Co-HNC-900, and Fe_1_Co-HNC-1000 all exhibit a distinct diffraction peak at about 26°, which can be attributed to (002) of graphitic carbon [29]. Fe_1_Co-HNC-900 and Fe_1_Co-HNC-1000 also demonstrate a diffraction peak at about 44.6°, which can be attributed to the Fe(110) crystal plane [30], indicating that Fe was successfully doped in the nitrogen-containing carbon composite. However, there is no obvious peak which can be ascribed to Co crystal using XRD as the low content of Co in the prepared composite (as given in Table S1). Hence, XPS was selected to further investigate the elemental composition and chemical state in the target catalyst. The results verified that the product consisted of C, N, O, Fe, and Co in Figure S4 and Table S2, proving the successful doping of Fe and Co. According to Figure 2a, the high-resolution C 1s XPS segment of Fe_1_Co-HNC-1000 can be divided into four sub-peaks at 288.5, 286.4, 285.2 and 284.7 eV, corresponding to O-C=O, C=O, C-O/C-N and C=C groups, respectively [31]. These abundant carbon–oxygen species are often considered as more active site-related defects that can promote ORR in alkaline environments [32]. The N 1s spectra of Fe_1_Co-HNC-1000 can be divided into four peaks in XPS, corresponding to pyridinic-N (398.5 eV), M-N_x_ (399. 7 eV, M = Fe, Co, and Zn), pyrrolic-N (400.5 eV), and graphitic-N (401.6 eV), as shown in Figure 2b. It was believed that the high contents of pyridinic-N and pyrrolic-N can provide high ORR activities [33,34]. Figure 2c,d display the XPS spectra of Co 2p and Fe 2p of Fe_1_Co-HNC-1000. The regional Co 2p spectrum in Figure 2c reveals a high energy band at 795.7 eV (Co 2p_1/2_) and a low energy band at 780.3 eV (Co 2p_3/2_), being in accordance with the report of Co_3_O_4_ [35]. And the energy bands at 722.1, 720.0, 709.8, and 707.5 eV are corresponding to Fe_3_O_4_ [20]. In addition, Figure S3 also exhibits the XRD patterns of Co-HNC-900, Fe_1_Co-HNC-900, and Fe_1_Co-HNC-1000. The peak at approximately 44.6° can be attributed to the (200) plane of Fe_3_O_4_ (Powder Diffraction File (PDF) # 28-0491) or the (400) plane of Co_3_O_4_ (PDF # 42-1467) [36,37]. Therefore, we can conclude that there is an oxidation state of Fe and/or Co in the prepared composite. Furthermore, the metal contents were also analyzed using MP-AES. According to Table S1, it is clear that Zn was attenuated as the increase in the pyrolysis temperature, and it was no longer detectable when the pyrolysis temperature was 1000 °C. Interestingly, when the temperature was constant, the added amount of Fe had little effect on the final loading amount of Co. At the same time, the M-N_x_ content increased from 10.4% (Co-HNC-900) to 15.32% (Fe_1_Co-HNC-900) with the addition of Fe (Figure 2e). It was believed that M-N_x_ are active sites which are responsible for the excellent electrocatalytic properties [38]. In addition, graphitic-N increased from 11.4% to 15.91%, whereas pyrrolic-N decreased from 16% to 5.45% as the temperature increased from 900 °C to 1000 °C (Figure 2e). The change should be attributed to that the unstable pyrrolic-N was converted to graphitic-N at high temperature, while graphitic-N can accelerate electron transfer and increase the limiting current density, thus favoring the enhancement of ORR activity [39]. Overall, the pyridinic-N content of Fe_1_Co-HNC-1000 is slightly higher than that of Fe_1_Co-HNC-900. It is believed that the metals Fe and Co are coordinated with pyridinic-N to form the active sites [40].

**Figure 3:**
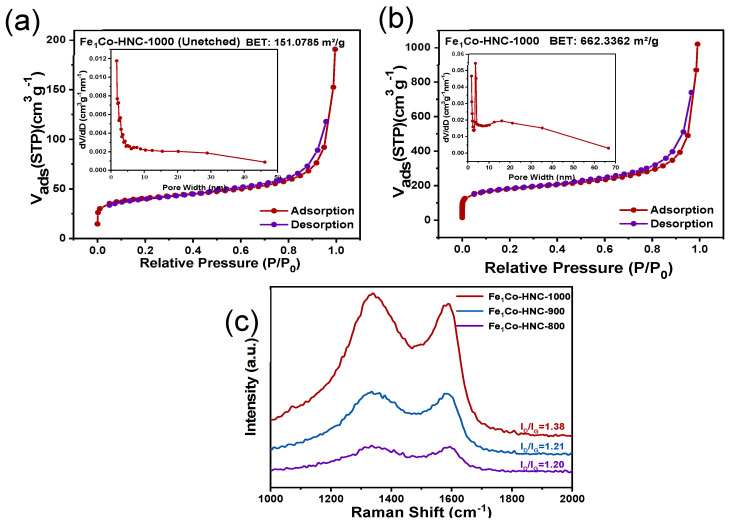
(**a**,**b**) N_2_ adsorption–desorption isotherms of Fe_1_Co-HNC-1000 (unetched) and Fe_1_Co-HNC-1000. (**c**) Raman spectra of Fe_1_Co-HNC were prepared at different temperatures.

The porous structure was investigated using the N_2_ adsorption–desorption method. The adsorption–desorption curves of the prepared composites all exhibited type IV isotherm curves which were consistent with the H4 hysteresis loop based on the N_2_ adsorption–desorption isotherms (Figure 3a,b and Table S3). The result exhibited that Fe_1_Co-HNC-1000 presents a high specific surface area of 662.33 m^2^ g^−1^ and an enormous total pore volume of 0.75 cm^3^ g^−1^. Compared with unetched-Fe_1_Co-HNC-1000 (151.07 m^2^ g^−1^), the specific surface area of Fe_1_Co-HNC-1000 was increased significantly. At the same time, as the temperature increased from 800 °C to 1000 °C, the average mesoporous diameter increased from 16.50 nm to 18.70 nm. The result indicated that higher temperature is more conducive to the formation of large mesopores. However, the average mesoporous diameter of the unetched-Fe_1_Co-HNC-1000 is only 14.88 nm, demonstrating that the effective removal of the silica template resulted in a larger mesoporous diameter and specific surface area. It was believed that the mesoporous structure can provide abundant active sites, which will facilitate mass transfer/electron transfer, thereby improving catalytic efficiency [41,42]. Based on Raman spectroscopy, defects and graphitization degree were estimated. The characteristic peaks are located at ≈1352 and ≈1602 cm^−1^ [43], respectively, corresponding to the D-band of defects and the G-band of sp^2^ graphitic carbon. According to Figure 3c, the ratio of defects increased with the increase in annealing temperature, which can be attributed to the Zn volatilization in the ZIF skeleton as the temperature increase. The sample prepared at 1000 °C has the largest I_D_/I_G_ (1.38), implying that there are abundant defects in Fe_1_Co-HNC-1000 which can contribute to the adsorption of reactants during the catalytic reaction.

### 2.2. Electrochemical Characterization

**Figure 4:**
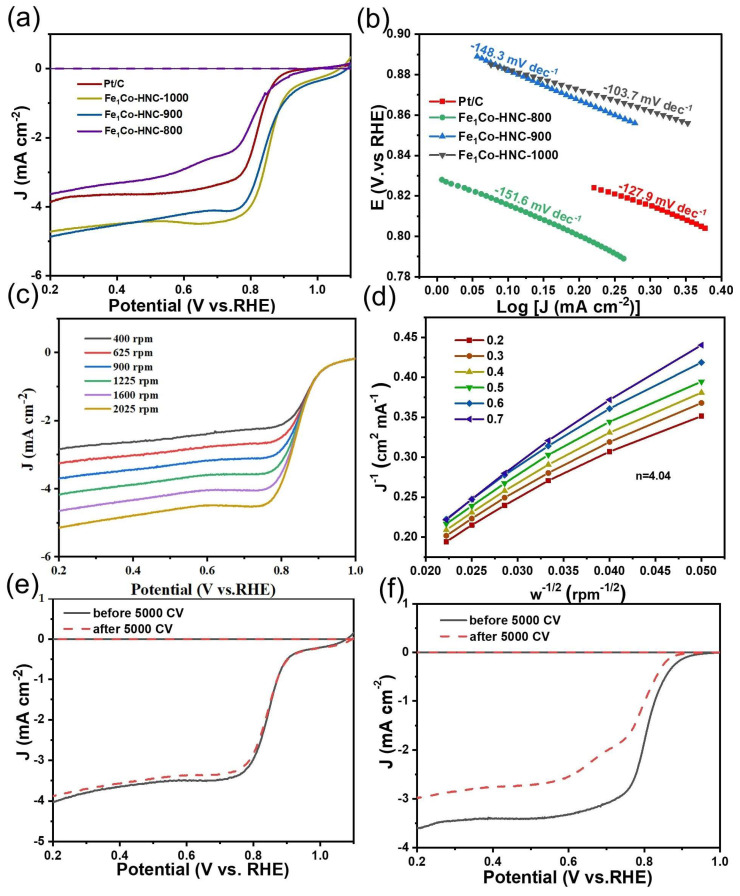
(**a**) RRDE polarization curves of different electrocatalysts. (**b**) Tafel slope plots. (**c**) LSV polarization curves of Fe_1_Co-HNC-1000 carried on various rotating rates. (**d**) Corresponding K-L plots and calculated n. (**e**) LSV curves of Fe_1_Co-HNC-1000, and (**f**) Pt/C in 0.10 M KOH before and after 5000 CV cycles. Scan rate is 5 mV/s.

The effect of the added amount of Fe on the ORR catalytic activity was checked at first. The linear sweep voltammetry (LSV) measurement was chosen to assess the electrocatalytic activity in oxygen-saturated 0.10 M KOH. The results indicated that the ORR half-wave potential (E_1/2_) of the monometallic Co doped Co-HNC-1000 was low (E_1/2_ = 0.78 V, Figure S5). As Fe was added, the ORR E_1/2_ shifted positively. When the Fe/Co ratio was 1:1, Fe_1_Co-HNC-900 demonstrated the highest E_1/2_ (0.85 V), indicating the effective synergistic effect between Fe and Co. Then, Fe_1_Co-HNC-1000 which was prepared at 1000 °C and the Fe:Co ratio of 1.0 exhibited the best ORR activity than those of other samples (Figure 4a and Table S4). The half-wave potential of Fe_1_Co-HNC-1000 is 0.86 V which is 10 mV more positive than that of Fe_1_Co-HNC-900. Moreover, the excellent ORR activity of Fe_1_Co-HNC-1000 is further reflected by the Tafel slope (Figure 4b), which is the smallest among all samples (103.7 mV dec^−1^), including the commercial Pt/C (127.9 mV dec^−1^). The result indicated that Fe_1_Co-HNC-1000 possessed a fast electron transfer rate for ORR. It can be ascribed to the unique 1D@2D structure, high content of pyridinic-N and graphitic-N, rich mesopores, plenty of defects, and the synergistic effect of Fe and Co [22,44,45].

To explore the ORR kinetics of Fe_1_Co-HNC-1000, LSV curves were obtained at different rotational speeds from 400 to 2025 rpm (Figure 4c), where J_L_ (J_L_ is the limiting current density) increased with increasing speed and fitted well with the first-order kinetic equations. From the Koutecky–Levich (K-L) plot in Figure 4d, it can be seen that the average electron transfer number (n) is about 4.06, reflecting the high efficiency of the direct reduction in oxygen to water on Fe_1_Co-HNC-1000. The hypothesis was further confirmed by rotating ring-disk electrode measurements (Figure S6), where the yield of H_2_O_2_ was 11% with an average n of 3.9, which were comparable with those of the commercial Pt/C.

The stability and methanol resistance of Fe_1_Co-HNC-1000 were analyzed and compared with those of commercial Pt/C. After 5000 CV cycles, the Fe_1_Co-HNC-1000 RRDE curve remained identical to the initial one (Figure 4e). In contrast, the RRDE curve of Pt/C exhibited an obvious negative shift after 5000 CV cycles, with E_1/2_ and J_L_ becoming significantly smaller (Figure 4f). Additionally, Fe_1_Co-HNC-1000 was subjected to extensive testing at 0.52 V for 30,000 s. The current density remained at 99.8% of the original one, which was much higher than the commercial Pt/C (85%), confirming that Fe_1_Co-HNC-1000 had excellent stability (Figure S7). The CV curves of Fe_1_Co-HNC-1000 for methanol were depicted in Figure S8a. There was no change before and after the addition of methanol for Fe_1_Co-HNC-1000, whereas Pt/C exhibited a significant methanol oxidation peak in the presence of 1.0 M methanol (Figure S8b). The result indicated that the active sites of Fe_1_Co-HNC-1000 have excellent tolerance to methanol, which is much superior to Pt/C. All of these results demonstrated that the Fe_1_Co-HNC-1000 catalyst is exceptionally durable and selective.

In addition, the electrochemically active surface area (ECSA) has a notable effect on the ORR [46], which can be assessed by the double-layer capacitance (C_dl_) in CV curves (Figure S9). Specifically, Fe_1_Co-HNC-1000 had the highest C_dl_ value of 43.36 mF cm^−2^, which implied a dramatic increase in ECSA of the 1D@2D Fe_1_Co-HNC-1000. It was hypothesized that Fe_1_Co-HNC-1000 exhibited a significantly increased charge transfer capability compared with its bimetallic counterpart prepared at lower temperatures, mainly due to the rich defects, mesoporous structure, and the unique 1D@2D structure. Notably, the C_dl_ value of the bimetallic catalyst was much higher than that of Co-HNC-900 (6.58 mF cm^−2^), which also highlighted the synergistic effect of Fe_1_Co-HNC-1000, creating more active sites. In addition, the electrochemical impedance spectra were selected to explain this phenomenon based on the interfacial charge transfer of the prepared catalysts, which can be calculated from the Nyquist diagram (Figure S10) [47]. The result displayed that Fe_1_Co-HNC-1000 had a smaller charge transfer resistance (R_ct_), which can produce faster ORR kinetics.

**Figure 5:**
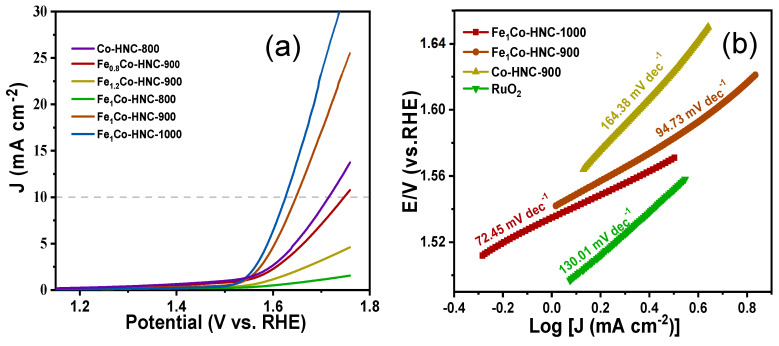
(**a**) RRDE polarization curves of different electrocatalysts at 1600 rpm. (**b**) Tafel slope plots. Scan rate is 5 mV/s.

The OER performance of different catalysts was tested and displayed in Figure 5. The overpotential of Fe_1_Co-HNC-1000 in 1.0 M KOH was 399 mV at the current density of 10 mA cm^−2^, which was slightly higher than that of RuO_2_ in 1.0 M KOH (388 mV). The difference between E_1/2_ of Fe_1_Co-HNC-1000 and its OER at J = 10 mA cm^−2^ was calculated to be 0.76 V, which is slightly lower than that of Pt/C + RuO_2_ (0.79 V). The result indicated the excellent bifunctional catalytic activity of Fe_1_Co-HNC-1000 (Table S5). In addition, the OER Tafel slope of Fe_1_Co-HNC-1000 (72.45 mV dec^−1^) was smaller than that of RuO_2_ (130.01 mV dec^−1^) according to the Tafel fitting curve as shown in Figure 5b. The result demonstrated that the kinetics of OER on Fe_1_Co-HNC-1000 is faster. Also, the OER Tafel slope of Fe_1_Co-HNC-1000 was lower than that of Fe_1_Co-HNC-900 (94.73 mV dec^−1^), indicating that Fe_1_Co-HNC-1000 has a faster mass transfer rate. The result is consistent with the BET results that the average mesopore diameter of Fe_1_Co-HNC-1000 is large, thus speeding up the diffusion rate. The high OER Tafel slope of Co-HNC-900 (164.38 mV dec^−1^) further indicates that there is a synergistic coupling formed between Fe and Co, and the existence of metal oxides should play the key role in improving OER catalytic activities [48,49].

In order to evaluate the effectiveness of Fe_1_Co-HNC-1000, it was employed as the air cathodic catalyst in an assembled zinc–air battery (Figure S11a). The open-circuit voltage of the battery was then measured using a multimeter (Figure S11b), yielding a value of 1.660 V for the Fe_1_Co-HNC-1000-modified cathode. In contrast, a zinc–air battery with Pt/C + RuO_2_ as the cathodic catalyst only produced an open-circuit voltage of 1.415 V. In addition, two zinc–air batteries with Fe_1_Co-HNC-1000 as the cathodic catalyst were successfully connected in series to light a small LED (Figure S11c). The power densities of the zinc–air batteries were also tested at different current densities, and the results showed that the zinc–air batteries with Fe_1_Co-HNC-1000 as the cathodic catalyst achieved the highest power density of 94.9 mW cm^−2^ at 159.8 mA cm^−2^, while the Pt/C + RuO_2_ catalyst achieved the highest power density of 80.3 mW cm^−2^ at 118.8 mA cm^−2^ (Figure S11d). This indicated that the Zn–air batteries with Fe_1_Co-HNC-1000 as the cathodic catalyst had higher electrochemical performance and power density than those of the Pt/C + RuO_2_ modified electrode. In order to verify its durability, a charge–discharge cycle test was carried out on zinc–air batteries assembled with Fe_1_Co-HNC-1000 or Pt/C + RuO_2_ as the cathodic catalyst (Figure S11e). The results indicated that Fe_1_Co-HNC-1000 outperformed Pt/C + RuO_2_ in terms of power density and durability, thus confirming the superior performance of Fe_1_Co-HNC-1000 in zinc–air batteries.

## 3. Experimental Section

### 3.1. Chemicals

2-methylimidazole (2-MIM) (98%), Fe(NO_3_)_3_·9H_2_O (99%), Co(NO_3_)_2_·6H_2_O (99%), HF (40%), n-hexanol (99%), and cyclohexane (99%) were supplied from Shanghai Aladdin Biochemical Technology Co., Ltd. (Shanghai, China). Triton X-100 (99%) and Nafion (5 wt%) were obtained by Sigma-Aldrich (St. Louis, USA). Commercially available Pt/C (20 wt%) was bought from Johnson Matthey Company (Shanghai, China). And RuO_2_ (99.9%), ethyl orthosilicate, 3-aminopropyltriethoxysilane (99%), NH_3_·H_2_O, polydiallyldimethylammonium chloride (PDDA, 99%), and sodium polystyrene sulfonate (PSS, 99%) were purchased from Shanghai McLean Biochemical Technology Co, Ltd. (Shanghai, China). All chemical reagents were used directly without further treatment.

### 3.2. Synthesis of Catalysts

#### 3.2.1. Preparation of Silica Nanospheres

The preparation of silica nanospheres was based on a modified typical reversed-phase microemulsion method [50]. The water/oil microemulsion was obtained by adding 15.4 mL of cyclohexane, 0.68 mL of pure water, 3.80 mL of Triton X-100, and 3.20 mL of hexanol to a 50 mL beaker and stirring uniformly for 30 min at room temperature. To the water/oil microemulsion system, 1.05 mL of ethyl orthosilicate was added and stirred for 5 h. Next, 0.35 mL of 3-aminopropyltriethoxysilane was added, and then 0.20 mL of NH_3_·H_2_O was added after stirring for 60 min at room temperature, to initiate the hydrolysis of ethyl orthosilicate and 3-aminopropyltriethoxysilane. After stirring for 18 h at room temperature, ethanol was added to destabilize the microemulsion system. Finally, the product was washed three times with ethanol, collected using centrifugation, and dried in a vacuum-drying oven.

#### 3.2.2. Preparation of Polyelectrolyte-Modified Silica Nanospheres (PP-SiO_2_)

The PDDA-modified silica nanospheres (P-SiO_2_) were obtained by adding 3.00 g of silica nanospheres to 200 mL of 1.0 wt% PDDA solution, stirring for 1 h at room temperature, washing with purified water and centrifuging three times, and drying overnight in a vacuum-drying oven. Similarly, the obtained P-SiO_2_ was treated with 200 mL of 1.0 wt% PSS solution, and PDDA-PSS-modified silica nanospheres (PP-SiO_2_) were obtained after washing with pure water, centrifugation, and drying, respectively [51].

#### 3.2.3. Preparation of Fe_x_Co-HNC-T

Preparation of solution I: PP-SiO_2_ (1.000 g), Zn(NO_3_)_2_·6H_2_O (1.119 g), and the molar ratio of Fe(NO_3_)_3_·9H_2_O to Co(NO_3_)_2_·6H_2_O was x/y (x is the mass of Fe(NO_3_)_3_·9H_2_O, y is the mass of Co(NO_3_)_2_·6H_2_O, and x/y = 0, 0.8, 1.0, or 1.2) to 40 mL of methanol and mixed thoroughly. Solution II was prepared by dissolving 2-methylimidazole (2.420 g) in 40 mL of methanol with 3.00 mL of NH_3_·H_2_O. Solution II was added to solution I drop by drop, stirred for 3 h, washed three times with methanol, then collected using centrifugation and placed under vacuum to dry overnight to obtain a brown powder. As a blank control group, ZIF-8/ZIF-67 was prepared under the same conditions (the molar ratio of doped Fe/Co was 1.0) in the absence of the functionalized PP-SiO_2_ template.

The above product was ground with an agate mortar and then placed in a tube furnace in nitrogen atmosphere for the following pyrolysis procedure: firstly, from room temperature to 240 °C at a heating rate of 2 °C min^−1^ and kept at 240 °C for 2 h, then from 240 to T °C (T = 800, 900, or 1000 ℃) at a heating rate of 2 °C min^−1^, and kept for 2 h. After cooling to room temperature, the silica template was etched with 10 wt % HF to obtain Fe_x_Co-HNC-T.

## 4. Conclusions

In conclusion, we have successfully developed a simple method to prepare a bifunctional catalyst with a Fe and Co bimetal-doped 1D CNTs and 2D CNSs composite. By precisely controlling the structure and composition of the precursors, it is possible to obtain electrocatalysts with open mesoporous structures and abundant active sites after pyrolysis. The catalysts exhibited excellent ORR and OER activities under alkaline conditions. In addition, the catalyst showed excellent performance in terms of open circuit potential and charge/discharge cycle life when it was applied to zinc–air batteries. The result offers the possibility of designing promising bifunctional catalysts to replace noble metal-based catalysts in the field of energy storage and conversion.

## Data Availability

The original contributions presented in the study are included in the article/Supplementary Materials, further inquiries can be directed to the corresponding authors.

## Supplementary Materials

The following supporting information can be downloaded at: https://www.mdpi.com/article/10.3390/molecules29102349/s1, Figure S1. SEM images of (**a**) SiO_2_, (**b**) PP-SiO_2_, (**c**) Fe/Co/Zn-ZIF-8/ZIF-67 without the addition of ammonia, and (**d**) Fe/Co/Zn-ZIF-8/ZIF-67 with the addition of ammonia. Figure S2. SEM images of (**a**) Fe_1_Co-HNC-1000 (un-etched, unmodified SiO_2_), (**b**) Co-HNC-900, and (**c**) Fe_1_Co@CNFs-900. Figure S3. XRD patterns of Co-HNC-900, Fe_1_Co-HNC-900, and Fe_1_Co-HNC-1000. Figure S4. XPS spectra of Fe_1_Co-HNC-1000. Figure S5. RRDE polarization curves of different electrocatalysts at 1600 rpm. Scan rate is 5 mV/s. Figure S6. H_2_O_2_ yield and n of (**a**) Fe_1_Co-HNC-1000, and (**b**) Pt/C. Figure S7. Chronoamperometric curves of Fe_1_Co-HNC-1000 and Pt/C at 0.52 V. Figure S8. CV curves of (**a**) Fe_1_Co-HNC-1000, and (**b**) Pt/C with and without 1.0 M CH_3_OH at O_2_-saturated 0.10 M KOH electrolyte. Scan rate is 5 mV/s. Figure S9. (**a**–**g**) CV curves of different electrocatalysts in 0.10 M KOH at various scan rate. (**a**) FeCo-NC-900, (**b**) Co-HNC-900, (**c**) Fe_0.8_Co-HNC-900, (**d**) Fe_1_Co-HNC-900, (**e**) Fe_1.2_Co-HNC-900, (**f**) Fe_0.8_Co-HNC-800, and (**g**) Fe_0.8_Co-HNC-1000. (**h**, **i**) Plots of ΔJ vs. scan rate at 1.064 V (vs. RHE) of different electrocatalysts in 0.10 M KOH. Figure S10. EIS of Fe_1_Co-HNC-900 and Fe_1_Co-HNC-1000 in 0.10 M KOH. Frequency range 100 kHz to 0.01 Hz, amplitude 5 mV, potential 0.52 V (vs. RHE). Figure S11. Electrochemical performance of the Fe_1_Co-HNC-1000 and commercial Pt/C + RuO_2_ mixture catalysts in a zinc–air battery. (**a**) Schematic illustration of the rechargeable liquid-state zinc–air battery. (**b**) Open-circuit potential plots (Illustrations: a: Fe_1_Co-HNC-1000 and b: Pt/C+RuO_2_ open circuit voltage diagram tested with a multimeter). (**c**) The photo of a lighted LED powered by two Fe_1_Co-HNC-1000-based zinc–air batteries in series. (**d**) Discharge polarization curves and power density plots. (**e**) Galvanostatic charge–discharge cycling curves at 10 mA cm^−1^. Table S1. Metal atom contents of each catalyst tested using MP-AES. Table S2. The elemental content of different samples obtained using XPS. Table S3. Pore parameters of Fe_1_Co-HNC synthesized at different temperatures. Table S4. Comparison of the ORR activity with different catalysts. Table S5. Comparison of the potential gap (ΔE) between ORR half-wave potential (^ORR^E_1/2_ = 0.86 V) and OER overpotential at 10 mA cm^−2^ (^OER^E_j_ = 10 mA) of Fe_1_Co-HNC-1000 with recently reported analogous Fe/Co-based electrocatalysts. References [52,53,54,55] are cited in the Supplementary Materials.
