# Supplementary material for: Fe-Co Co-Doped 1D@2D Carbon-Based Composite as an Efficient Catalyst for Zn–Air Batteries
Source: Molecules. 2024 May 16;29(10):2349. doi: 10.3390/molecules29102349 (PMC11123740; doi:10.3390/molecules29102349)
Supplement: Supplementary file 1 [file molecules-29-02349-s001.zip › molecules-2994669-supplementary.pdf]

Supplementary materials

Fe-Co Co-Doped 1D@2D Carbon-Based Composite as an Efficient Catalyst for Zn–Air Batteries

Ziwei Deng [1], Wei Liu [2], Junyuan Zhang [1], Shuli Bai [1], Changyu Liu [1], Mengchen Zhang [1], Chao Peng [1], Xiaolong Xu [1,*] and Jianbo Jia [1,*]

[1] Jiangmen Key Laboratory of Synthetic Chemistry and Cleaner Production, School of Environmental and Chemical Engineering, Carbon Neutrality Innovation Center, Wuyi University, Jiangmen 529020, China
[2] Jiangmen Customs District Technology Center, Jiangmen 529020, China
* Correspondence: xuxl@wyu.edu.cn (X.X.); jbjiagu@163.com (J.J.)

Sample Characterization

The X-ray powder diffraction (XRD) patterns were tested on the Bruker D8 ADVANCE A25X diffractometer by using Cu Kα radiation (λ = 0.15406 nm). The morphological features of the samples were tested using a scanning electron microscope (SEM, GeminiSEM 300 German). TEM (TEM, FEI Talos-F200S, US) obtained clearer internal morphology and structure of carbon nanotubes and carbon nanosheets. The Brunel–Emmett–Taylor (BET) surface area and pore size distribution of Fe_xCo@NC-T were recorded and computed using an automated surface area and pore size analyzer (Micromeritics ASAP 2460, VacPrep 061, USA). The surface elements and valence were determined using an X-ray photoelectron spectroscopy (XPS) analyzer (Thermo Scientific K-Alpha, USA), and the obtained spectra were also compared with the carbon peak (284.8 eV) of the carbon monomer calibration. Raman spectrum was obtained at room temperature using a WITec alpha300R with an excitation wavelength of 532 nm. The metal content was tested with an Agilent CrossLab 4210 microwave plasma atomic emission spectrometer (MP-AES, USA) with emission wavelengths of Fe: 371.993 nm, Co: 340.512 nm, and Zn: 213.857 nm, respectively.

Electrochemical Test

All electrochemical tests were performed on a CHI760E electrochemical workstation with the conventional three-electrode system, i.e., a catalyst-loaded glassy carbon electrode as the working electrode, an Ag/AgCl (saturated KCl) electrode as the reference electrode, and a graphite rod as the counter electrode. The prepared electrocatalyst or commercially available 20 wt.% Pt/C was dispersed in 2.0 mL of mixture of water: isopropanol: naphthol = 21:21:0.075 (V/V), sonicated for 1.5 h, and then dropped onto the glassy carbon electrode and dried under an infrared lamp. The ring-disk electrode has a ring electrode area of 0.188 cm^2 and a disk electrode area of 0.126 cm^2. The loading of the prepared electrocatalyst in the ORR test was 1.0 mg cm^{-2}, and the loading of the commercially available Pt/C was 25 μg_{Pt} cm^{-2}. The ORR tests in alkaline media were performed in 0.10 M KOH saturated with O_2. The Rotating ring-disk electrode (RRDE) techniques were performed on a Model RRDE-3A Apparatus (ALS, Japan) in the potential range of 1.162 ~ 0.162 V (vs. reversible hydrogen electrode, RHE) at a sweep speed of 5 mV s^{-1}, and the ring potential was 1.162 V in alkaline media, respectively. All the potential conversion equation is E (vs. RHE) = E (vs. Ag/AgCl) + 0.059pH + 0.197 V.

The formula for H_2O_2 yield (H_2O_2%) and electron transfer (n) is as follows:

$$H_2O_2\% = \frac{200\frac{I_r}{N}}{I_d + \frac{I_r}{N}} \quad (1)$$

$$n = \frac{4I_d}{I_d + \frac{I_r}{N}} \quad (2)$$

Where N is the current collection efficiency (calculated as 0.44), I_d and I_r are the disk and ring currents, respectively.

The ORR linear sweep voltammetry (LSV) curves at various speeds were recorded to calculate n based on the Kentucky–Levich equation in the ORR process.

$$\frac{1}{J} = \frac{1}{J_L} + \frac{1}{J_K} = \frac{1}{B\omega^{1/2}} + \frac{1}{J_K} \quad (3)$$

$$B = 0.2nFC_0(D_0)^{2/3}V^{-1/6} \quad (4)$$

Zn–Air Battery Assembly and Testing

To assemble the Zn–air battery, a polished zinc plate (0.3 mm of thickness) was used as the anode; an air electrode coated with 1.25 mL catalyst ink of Fe_1Co-HNC-1000 or a mixture of 20 wt% Pt/C + RuO_2 (1:1 in a mass ratio) onto a carbon paper (electrode area: 12.5 cm^2; catalyst loading: 0.10 mg cm^{-2}). The air electrode was dried to form a uniform catalyst layer. In total, 6.0 M KOH with 0.20 M zinc acetate was used as the electrolyte for the rechargeable Zn–air batteries. The time for each charge and discharge in a cycle is 500 s. The potential–current polarization curves for the batteries were recorded on a CHI 660E workstation.

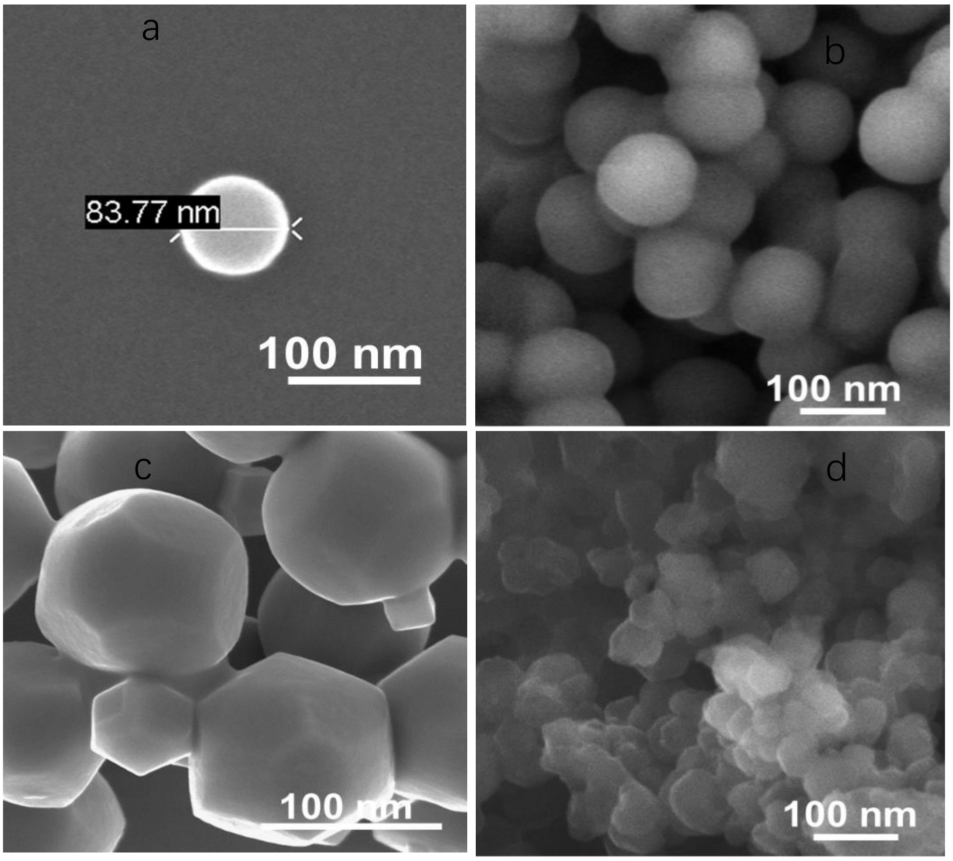

Figure S1. SEM images of (**a**) SiO_2, (**b**) PP-SiO_2, (**c**) Fe/Co/Zn-ZIF-8/ZIF-67 without the addition of ammonia, and (**d**) Fe/Co/Zn-ZIF-8/ZIF-67 with the addition of ammonia.

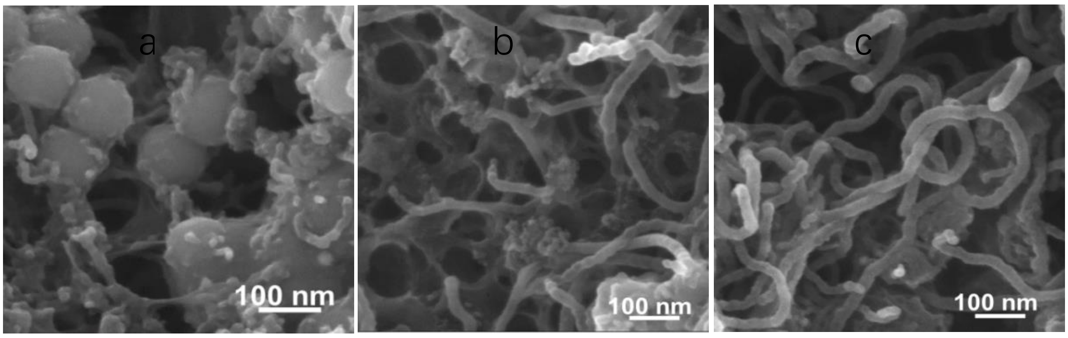

Figure S2. SEM images of (**a**) Fe_1Co-HNC-1000 (un-etched, unmodified SiO_2), (**b**) Co-HNC-900, and (**c**) Fe_1Co@CNFs-900.

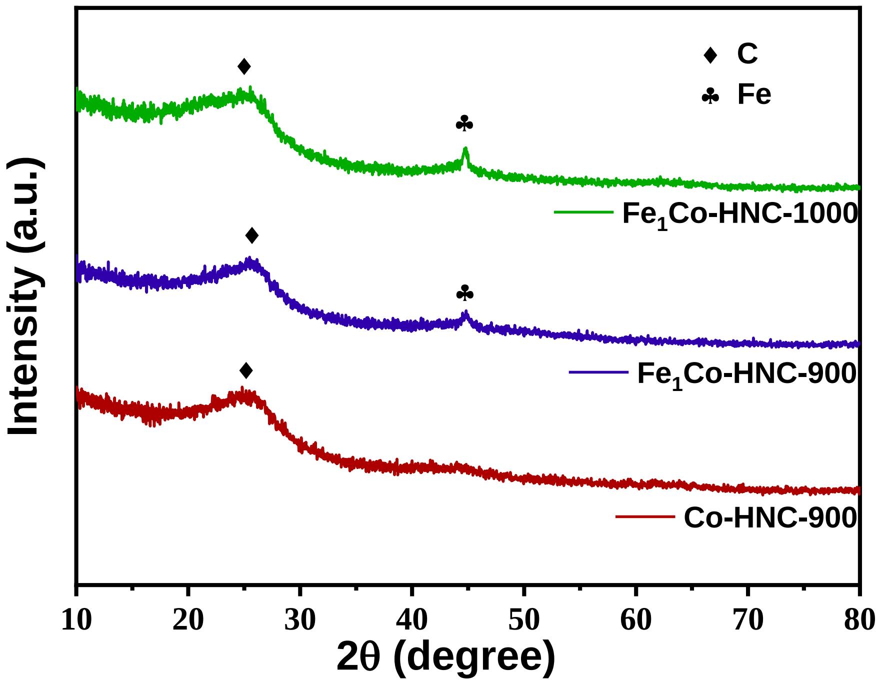

Figure S3. XRD patterns of Co-HNC-900, Fe_1Co-HNC-900, and Fe_1Co-HNC-1000.

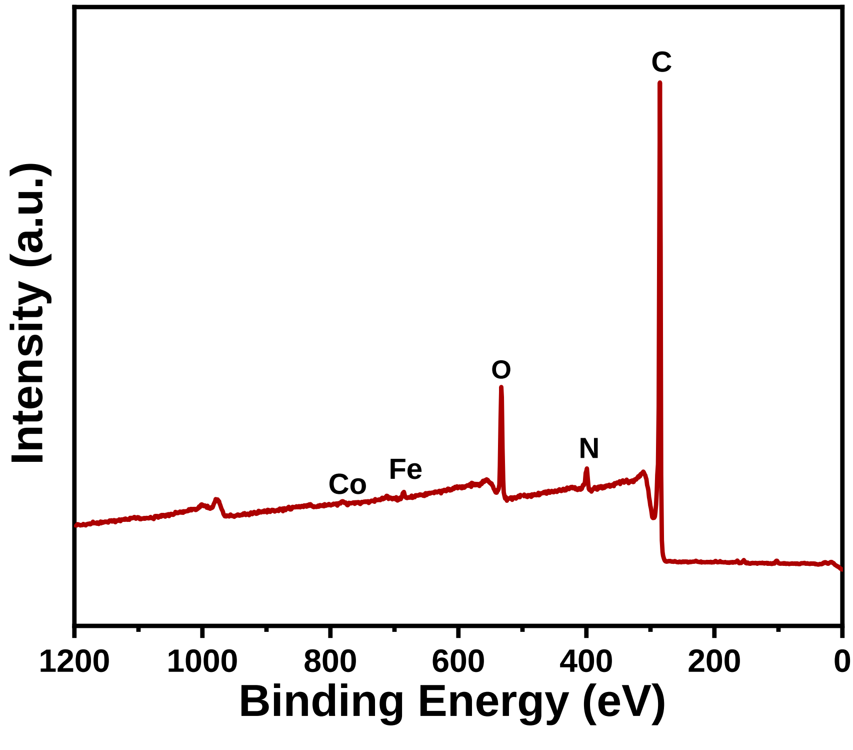

Figure S4. XPS spectra of Fe_1Co-HNC-1000.

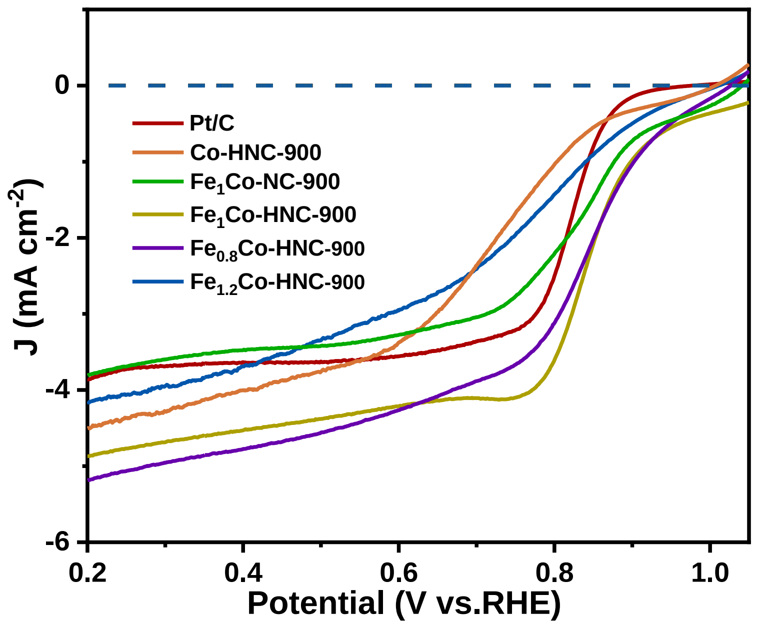

Figure S5. RRDE polarization curves of different electrocatalysts at 1600 rpm. Scan rate is 5 mV/s.

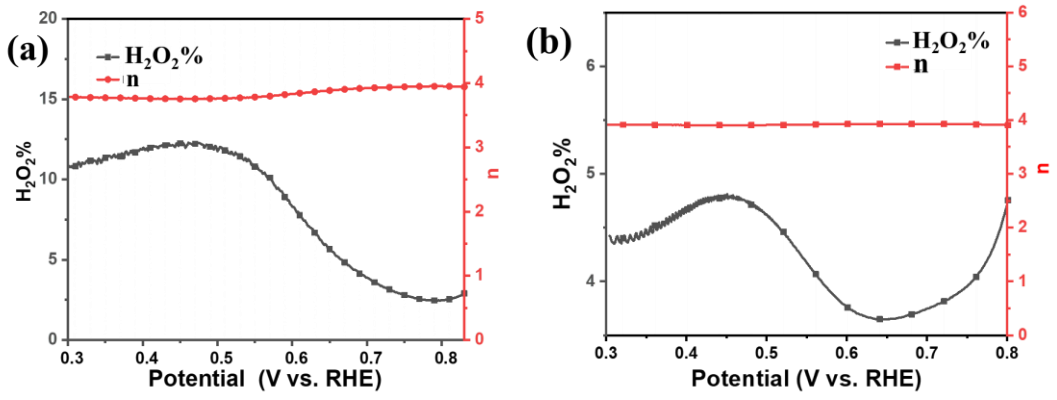

Figure S6. H_2O_2 yield and n of (**a**) Fe_1Co-HNC-1000, and (**b**) Pt/C.

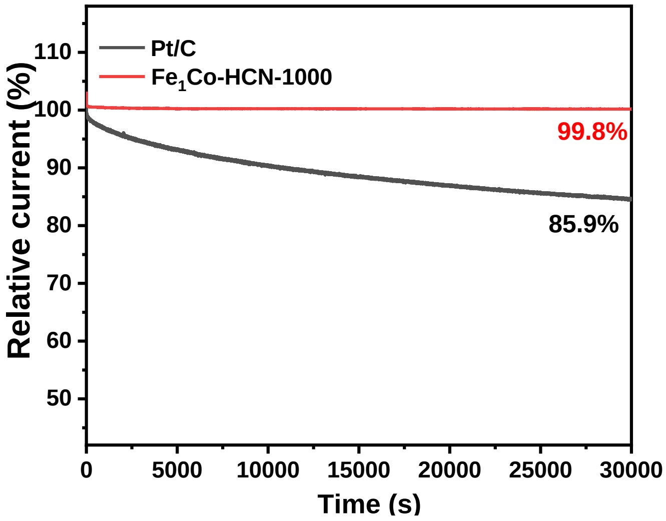

Figure S7. Chronoamperometric curves of Fe_1Co-HNC-1000 and Pt/C at 0.52 V.

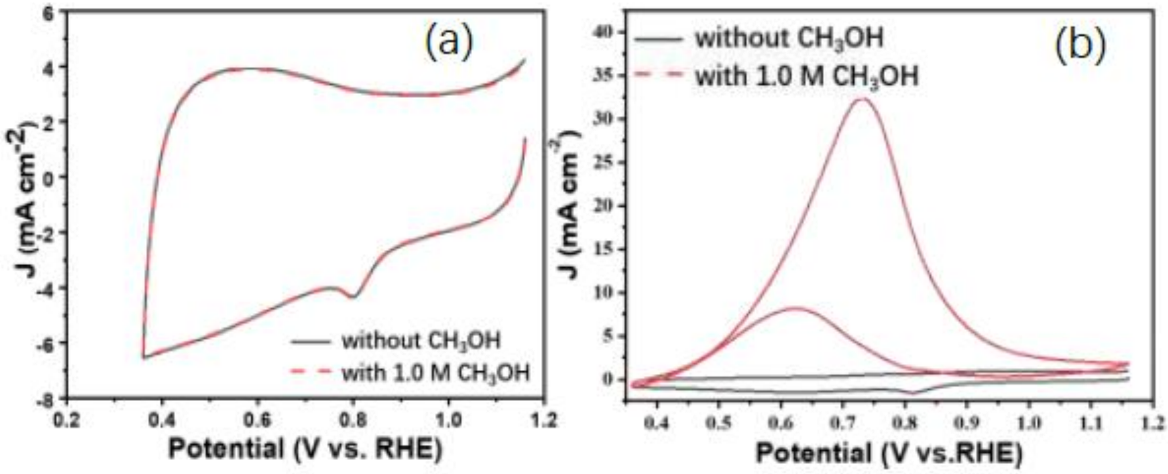

Figure S8. CV curves of (**a**) Fe_1Co-HNC-1000, and (**b**) Pt/C with and without 1.0 M CH_3OH at O_2-saturated 0.10 M KOH electrolyte. Scan rate is 5 mV/s.

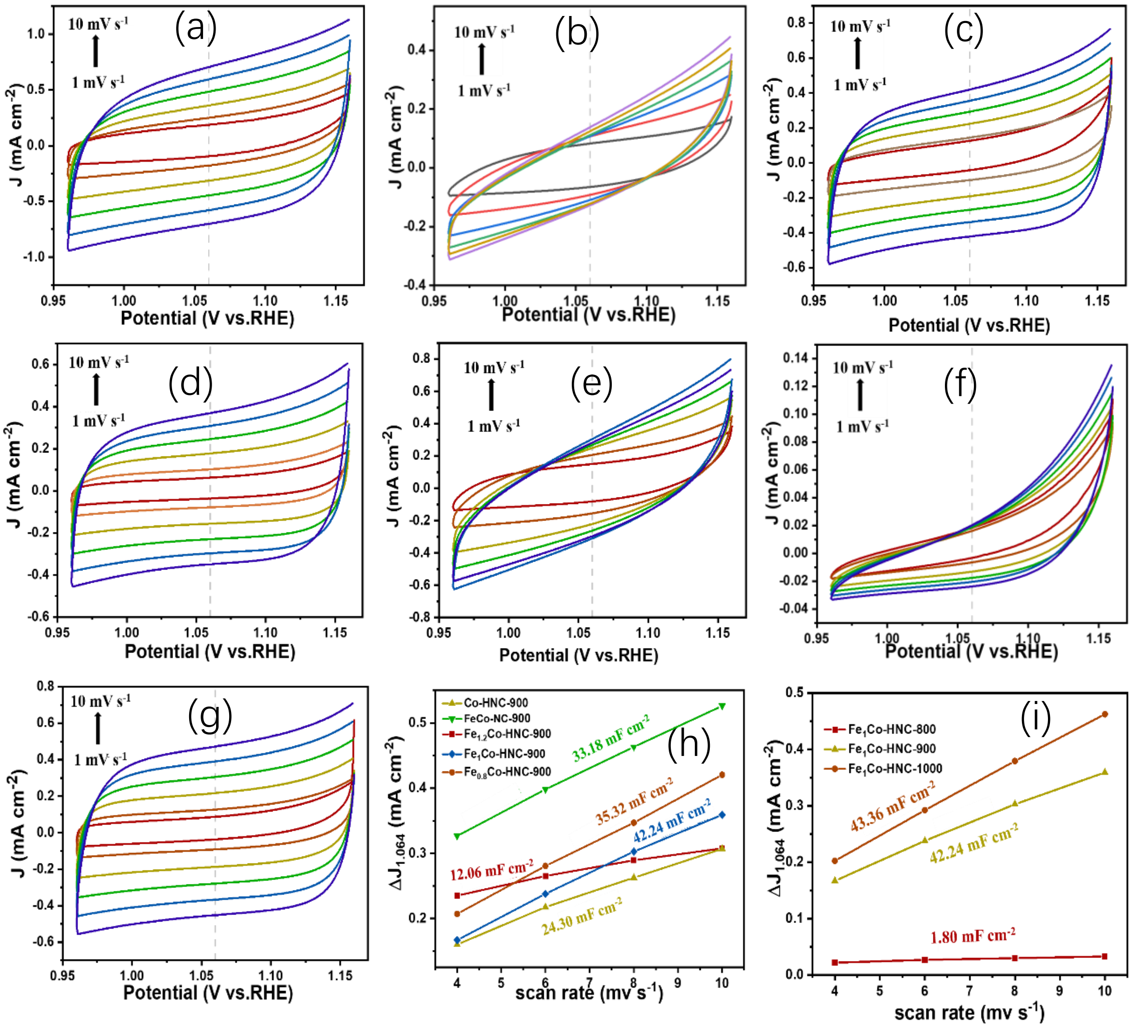

Figure S9. (**a**–**g**) CV curves of different electrocatalysts in 0.10 M KOH at various scan rate. (**a**) FeCo-NC-900, (**b**) Co-HNC-900, (**c**) $Fe_{0.8}Co$-HNC-900, (**d**) Fe_1Co-HNC-900, (**e**) $Fe_{1.2}Co$-HNC-900, (**f**) $Fe_{0.8}Co$-HNC-800, and (**g**) $Fe_{0.8}Co$-HNC-1000. (h, i) Plots of ΔJ vs. scan rate at 1.064 V (vs. RHE) of different electrocatalysts in 0.10 M KOH.

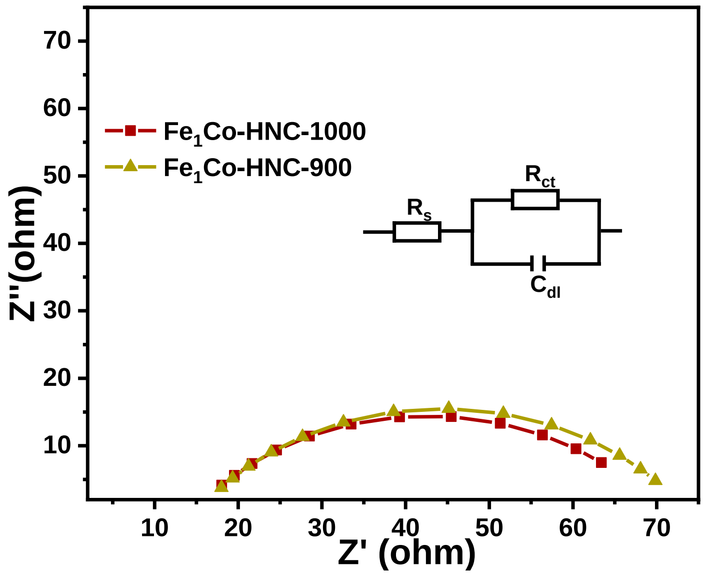

Figure S10. EIS of Fe_1Co-HNC-900 and Fe_1Co-HNC-1000 in 0.10 M KOH. Frequency range 100 kHz to 0.01 Hz, amplitude 5 mV, potential 0.52 V (vs. RHE).

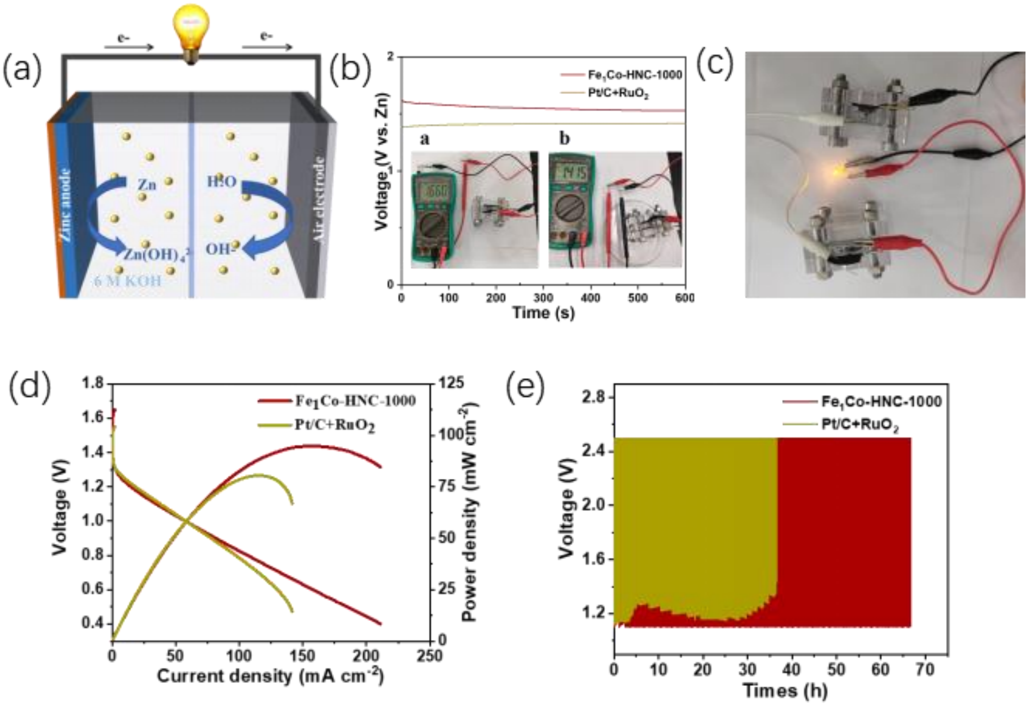

Figure S11. Electrochemical performance of the Fe_1Co-HNC-1000 and commercial Pt/C + RuO_2 mixture catalysts in a zinc–air battery. (**a**) Schematic illustration of the rechargeable liquid-state zinc–air battery. (**b**) Open-circuit potential plots (Illustrations: a: Fe_1Co-HNC-1000 and b: Pt/C+RuO_2 open circuit voltage diagram tested with a multimeter). (**c**) The photo of a lighted LED powered by two Fe_1Co-HNC-1000-based zinc–air batteries in series. (**d**) Discharge polarization curves and power density plots. (**e**) Galvanostatic charge–discharge cycling curves at 10 mA cm^{-1}.

Table S1. Metal atom contents of each catalyst tested using MP-AES.

Catalyst	Fe (at.%)	Co (at.%)	Zn (at.%)
Co-HNC-900	-	0.008	0.27
Fe_1Co-NC-900	0.029	0.004	2.21
$Fe_{0.8}Co$-HNC-900	0.217	0.003	0.05
Fe_1Co-HNC-900	0.165	0.003	0.21
$Fe_{1.2}Co$-HNC-900	0.261	0.003	0.22
Fe_1Co-HNC-1000	0.102	0.001	-

-: Not detected.

Table S2. The elemental content of different samples obtained using XPS.

Catalyst	C (at.%)	N (at.%)	O (at.%)	Zn (at.%)	Fe (at.%)	Co (at.%)
Fe_1Co-NC-900	78.69	9.19	10.26	1.07	0.1	0.69
Co-HNC-900	80.39	9.66	9.01	0.31	-	0.63
$Fe_{0.8}Co$-HNC-900	83.59	6.46	9.51	0.08	0.12	0.24
Fe_1Co-HNC-900	85.25	6.16	8.08	0.13	0.15	0.23
$Fe_{1.2}Co$-HNC-900	82.28	7.67	9.34	0.07	0.17	0.46
Fe_1Co-HNC-1000	85.56	4.31	9.96	-	0.07	0.10

-: Not detected.

Table S3. Pore parameters of Fe_1Co-HNC synthesized at different temperatures.

Catalyst	S_{BET} (m^2 g^{-1})	S_{mic} (m^2 g^{-1})	V_{tot} (cm^3 g^{-1})	Average pore diameter (nm)	Average mesopore diameter (nm)
Fe_1Co-HNC-800	536.89	313.15	0.53	4.01	16.50
Fe_1Co-HNC -900	485.76	212.79	0.61	5.07	17.25
Fe_1Co-HNC -1000	662.33	316.27	0.75	4.57	18.70
Fe_1Co-HNC-1000 (Unetched)	151.07	90.89	0.142	3.77	14.88

Table S4. Comparison of the ORR activity with different catalysts.

Catalyst	E_{onset} (V)	$E_{1/2}$ (V)	J_L (mA cm^{-2})	C_{dl} (mF cm^{-2})
Pt/C	0.95	0.83	3.85	
Co-HNC-900	0.89	0.76	4.50	24.30
Fe_1Co-NC-900	0.92	0.84	3.81	33.18
$Fe_{0.8}Co$-HNC-900	0.94	0.85	5.18	35.32
Fe_1Co-HNC-900	0.95	0.85	4.87	42.24
$Fe_{1.2}Co$-HNC-900	0.95	0.79	4.16	12.06
Fe_1Co-HNC-800	0.94	0.82	3.63	1.80
Fe_1Co-HNC-1000	0.96	0.86	4.72	43.36

Table S5. Comparison of the potential gap (ΔE) between ORR half-wave potential ($^{ORR}E_{1/2}$ = 0.86 V) and OER overpotential at 10 mA cm^{-2} ($^{OER}E_j$ = 10 mA) of Fe_1Co-HNC-1000 with recently reported analogous Fe/Co-based electrocatalysts.

NO.	Name	Category	ORR ($E_{1/2}$) (V)	OER (E_J = 10) (V)	△E (V)	Ref.
1	Fe/Co/N–C	3D@graphene	0.850	-	-	[1]
2	Fe, Co/N-C	FeCo nanoalloys encapsulated in N-doped carbon nanofibers	0.860	1.618	0.758	[2]
3	Co_3Fe_7@Co/Fe -SAC	N-doped carbon nanofiber networks	0.841	-	-	[3]
4	FeCo-N	3D multi-layered graphene structure with multi-channels	0.860	1.620	0.76	[4]
5	Pt/C + RuO_2/C	Precious metal catalysts	0.830	1.620	0.79	This work
6	Fe_1Co-HNC-1000	1D@2D	0.860	1.629	0.77	This work

-: not provided.

References

1. Xiong J, Chen X, Zhang Y, Lu Y, Liu X, Zheng Y, Lin J, Fe/Co/N–C/graphene derived from Fe/ZIF-67/graphene oxide three dimensional frameworks as a remarkably efficient and stable catalyst for the oxygen reduction reaction[J]. *RSC Advances*, **2022**, 12: 2425.
2. Yang W, Guo J, Ma J, Wu N, Xiao J, Wu M, FeCo nanoalloys encapsulated in N-doped carbon nanofibers as a trifunctional catalyst for rechargeable Zn-air batteries and overall water electrolysis[J]. *Journal of Alloys and Compounds*, **2022**, 926: 166937.
3. Liu B, Wang S, Feng R, Ni Y, Song F, Liu Q, Anchoring bimetal single atoms and alloys on N-doping-carbon nanofiber networks for an efficient oxygen reduction reaction and zinc–air batteries[J]. *ACS Applied Materials & Interfaces*, **2022**, 14: 38739.
4. Ji C, Zhang T, Sun P, Li P, Wang J, Zhang L, Sun Y, Duan W, Li Z, Facile preparation and properties of high nitrogen-containing Fe/Co/N co-doped three-dimensional graphene bifunctional oxygen catalysts for zinc air battery, *International Journal of Hydrogen Energy*, **2023**, 48, 26328–26340.
